# Beta-lapachone inhibits pathological retinal neovascularization in oxygen-induced retinopathy *via* regulation of HIF-1α

**DOI:** 10.1111/jcmm.12235

**Published:** 2014-02-18

**Authors:** Sung Wook Park, Jin Hyoung Kim, Ko-Eun Kim, Moon Hee Jeong, Hyunsung Park, Bongju Park, Young-Ger Suh, Woo Jin Park, Jeong Hun Kim

**Affiliations:** a Fight against Angiogenesis-Related Blindness Laboratory, Clinical Research Institute, Seoul National University HospitalSeoul, Korea; b Department of Biomedical Sciences, College of Medicine, Seoul National UniversitySeoul, Korea; c Department of Ophthalmology, Seoul National University HospitalSeoul, Korea; d Global Research Laboratory and Life Sciences Concentration, Gwangju Institute of Science and Technology (GIST)Gwangju, Korea; e Department of Life Science, University of SeoulSeoul, Korea; f College of Pharmacy, Seoul National UniversitySeoul, Korea

**Keywords:** β-lapachone, hypoxia-induced factor 1-α, oxygen-induced retinopathy, retinal neovascularization, retinopathy of prematurity, vascular endothelial growth factor

## Abstract

Retinal neovascularization in retinopathy of prematurity (ROP) is the most common cause of blindness for children. Despite evidence that hypoxia inducible factor (HIF)-1α -VEGF axis is associated with the pathogenesis of ROP, the inhibitors of HIF-1α have not been established as a therapeutic target in the control of ROP pathophysiology. We investigated the hypothesis that degradation of HIF-1α as a master regulator of angiogenesis in hypoxic condition, using β-lapachone, would confer protection against hypoxia-induced retinopathy without affecting physiological vascular development in mice with oxygen-induced retinopathy (OIR), an animal model of ROP. The effects of β-lapachone were examined after intraocular injection in mice with OIR. Intraocular administration of β-lapachone resulted in significant reduction in hypoxia-induced retinal neovascularization without retinal toxicity or perturbation of developmental retinal angiogenesis. Our results demonstrate that HIF-1α–mediated VEGF expression in OIR is associated with pathological neovascularization, not physiological angiogenesis. Thus, strategies blocking HIF-1α in the developing eye in the pathological hypoxia could serve as a novel therapeutic target for ROP.

## Introduction

Angiogenesis is a complex process to form new blood vessels, and essential for the organ growth in the development and the repair in disease [1]. Pathological angiogenesis in the retina, however, is a major cause of vision loss in retinopathy of prematurity (ROP), diabetic retinopathy and age-related macular degeneration (AMD) in each age group. Among angiogenesis-related blindness, ROP occurs through the partial regression of pre-existing vessels by vaso-obliteration followed by the pathological angiogenesis in developing retinal vasculature with relative hypoxia [2]. Thus, for the treatment of ROP, it is necessary to inhibit pathological angiogenesis while promoting normal physiological angiogenesis.

The oxygen-induced retinopathy (OIR) as an animal model of ROP simulates the vaso-obliterative and neovascularization phases of ROP with high reproducibility and, in turn, enables to assess treatment outcome of anti-angiogenic drugs. In addition, the retinal angiogenesis in the OIR mice enables to approach both physiological retinal vascular development and pathological neovascularization under pathological condition.

Among the molecular mechanism relevant to the pathogenesis of ROP, hypoxia inducible factor (HIF)-1α is the important oxygen-dependent regulator in both the vaso-obliterative phase where HIF-1α is suppressed and the neovascularization phase where HIF-1α produces angiogenic factors. Furthermore, loss of hypoxic response and VEGF production in astrocyte does not impair normal retinal vascular development [3]. Thus, it has been suggested that regulating HIF-1α could inhibit the pathological neovascularization without affecting physiological angiogenesis. Currently, most anti-angiogenic drugs used in ocular neovascular diseases target VEGF for the treatment [4,5]. However, VEGF acts as not only an angiogenic factor but also a neurotrophic factor in the development. Thus, despite current anti-VEGF treatment had good clinical results on ROP, new therapeutic target is strongly required to avoid potential adverse effect by targeting VEGF directly for the treatment of ROP [6].

During our efforts on developing anti-angiogenic drugs [7–12], we recently found that β-lapachone (3,4-dihydro-2,2-dimetyl-2H-naphthol[1,2-b]pyran-5,6-dione) has a good activity to regulate HIF-1α and elicits anti-angiogenic effect. This quinone-containing compound, a substrate of NQO1 (NAD(P)H:quinone oxidoreductase, has shown anti-ageing as well as anti-cancer activities [13].

In this study, we demonstrated that β-lapachone effectively reduced retinal neovascularization in OIR without toxicity. The anti-angiogenic effect of β-lapachone was related to HIF-1α degradation and subsequent attenuation of VEGF expression. Importantly, β-lapachone–targeting HIF-1α could inhibit pathological retinal neovascularization in OIR without disturbing physiological retinal angiogenesis in the development. Taken together, our results suggest that β-lapachone–targeting HIF-1α has a therapeutic potential as an anti-angiogenic drug for the ischaemic retinopathy.

## Materials and methods

### Animals

C57BL/6J mice were purchased from Central Lab. Animal Inc. (Seoul, Korea). Care, use and treatment of all animals in this study were in strict agreement with the ARVO statement for the Use of Animals in Ophthalmic and Vision Research. C57BL/6J mice were kept in standard 12-hr dark–light cycle and ∼23°C room temperature.

### Cell cultures

Human retina microvascular endothelial cells (HRMECs) were purchased from the Applied Cell Biology Research Institute (Kirkland, WA, USA) and were grown in a gelatin- coated 75-cm^2^ flask in an M199 medium (Gibco BRL, Carlsbad, CA, USA) supplemented with 20% foetal bovine serum (Gibco BRL), 3 ng/ml basic fibroblast growth factor (Millipore, Bedford, MA, USA) and 10 U/ml heparin (Sigma-Aldrich, St. Louis, MO, USA) at 37°C in an incubator with a humidified atmosphere of 95% O_2_ and 5% CO_2_. The experiments were performed with cells between passages 5 and 9. Human brain astrocytes were purchased from the Applied Cell Biology Research Institute and were grown in DMEM (Thermo scientific Hyclone, Logan, UT, USA) supplemented with 20% foetal bovine serum. The experiments were performed with cells between passages 10 and 16.

### Oxygen-induced retinopathy

With some modifications [12], OIR was induced as described by Smith *et al*. [14]. Briefly, newborn C57BL/6J mice were randomly assigned to two experimental (*n* = 6 respectively) and control (*n* = 6) groups. At post-natal day (P) 7, mice pups were subjected to hyperoxia (75 ± 0.5% oxygen) for 5 days (from P7 to P12) and return to room air (21% oxygen) for 5 days. Neovascularization occurs upon return to room air and peaks at P17. Oxygen was checked twice daily with an oxygen analyser (Miniox I; Bertocchi srl Elettromedicali, Cremona, Italy). To assess the anti-angiogenic activity of β-lapachone, pups were injected 1 μl intravitreously with 1 μM β-lapachone on P14, when retinal neovascularization began. These experiments were repeated at least three times.

### Qualitative assessment of retinal neovascularization by fluorescein angiography

As our previous description [12], at P17, deeply anesthetized OIR mice were intracardially injected with a fluorescein-conjugated dextran (molecular weight = 500,000; Sigma-Aldrich) dissolved in PBS. After 1-hr perfusion, the eyes were enucleated and fixed in 4% paraformaldehyde for 1 hr. The retinas were dissected, flat-mounted and viewed by a fluorescein microscopy (BX50; Olympus, Tokyo, Japan) at a magnification of 40×.

### Quantitative assessment of retinal neovascularization

As our previous description [12], at P17, the eyes were removed from mice with OIR, fixed in 4% paraformaldehyde for 24 hrs and embedded in paraffin. Sagittal sections of 4 μm, each 30 μm apart, were cut through the cornea parallel to the optic nerve. The sections were stained with haematoxylin and eosin to assess the vascular lumens of new vessels growing into the vitreous *via* light microscopy (Carl zeiss, Chester, VA, USA). Vascular lumens between posterior lens capsule and the inner limiting membrane were counted in at least 10 sections from each eye (at least five on each side of the optic nerve) by two independent observers blinded to treatment. The average number of intravitreal vessels per section was calculated for each group.

### Cell viability assay

Cell viability was determined by the 2-(4-iodophenyl)-3-(4-nitrophenyl)-5-(2, 4-disulfophenyl)-2H-tetrazolium, monosodium salt (WST-1) assay according to the manufacturer*s instructions [15]. HRMECs (1 × 10^4^ cells) were seeded into each well of 96-well plates with 100 μl of media. After incubation for 24 hrs, cells were treated with either dimethyl sulfoxide (DMSO) as a control or various concentration of β-lapachone (0.1–5 μM) for 48 hrs. Then, 10 μl of WST-1 solution (Daeil Lab Service Co. Ltd, Seoul, Korea) was added into each well and incubated at 37°C for 2 hrs. Absorption at 450 nm was measured using a microplate spectrophotometer (Molecular Devices, Sunnyvale, CA, USA). Absorption at 630 nm was measured as a background. Three independent experiments were performed for each experimental condition.

### Histological analysis

To evaluate the retinal toxicity of β-lapachone, 1 μl of 3 μM β-lapachone was injected intravitreously to 6-week-old male C57BL/6J mice. The mice were killed and the eyes were enucleated at 1 and 7 days after injection. Enucleated globes were fixed in 4% paraformaldehyde for 24 hrs, and embedded in paraffin. Retina was evaluated 1 and 7 days after intravitreous injection respectively. Haematoxylin and eosin stain was performed for histological examination. For the evaluation of changes in the retinal layers, the ratio of the retinal thickness from the internal limiting membrane to the inner nuclear layer to the retina thickness from the internal limiting membrane to the outer nuclear layer was measured and compared with those of control mice, as previously described [16].

### Terminal deoxynucleotidyl transferase biotin-dUTP nick end labelling (TUNEL) assay

Retinal cell apoptosis was evaluated 1 and 7 days after intravitreous injection of 3 μM β-lapachone in 6-week adult mice respectively. Immunofluorescence stain was performed with cleave caspase-3 (rabbit anti-cleaved caspase-3, 1:1000; Cell Signaling, Danvers, MA, USA) and TUNEL staining was performed with the Roche In Situ Cell Death Detection TMR red kit (Roche Diagnostic, Indianapolis, IN, USA) following the manufacturer*s instructions. Cleaved caspase-3–positive and TUNEL-positive cells were evaluated in three randomly selected fields (400×) under fluorescein microscopy (BX50; Olympus).

### Western blotting

Western blotting was performed with standard western blotting methods. Human brain astrocytes were incubated with 1 μM β-lapachone for 4 hrs under co-treatment of 125 μM CoCl_2_ or hypoxia (3% O_2_). Cell proteins were extracted with radioimmunoprecipitation assay buffer (Tris 50 mM pH 7.4; NaCl 150 mM; SDS 0.1%; NaDeoxycholate 0.5%; Triton X-100 1%; Cell Signaling Technology) with a complete protease inhibitor cocktail (Roche Molecular Biochemicals, Indianapolis, IN, USA) and incubated on ice for 1 hr and centrifuged at 20,000 × g for 30 min. at 4°C. The protein concentration was measured using a BCA protein assay kit (Pierce, Rockford, IL, USA). Equal amounts of protein from the supernatant were separated by SDS-PAGE using 6–12% Tris-Tricine gel (Bio-Rad Laboratories Inc., Hercules, CA, USA) and transferred to nitrocellulose membranes (Amersham Hybond ECL; GE healthcare, Piscataway, NJ, USA). The membranes were blocked in PBS with 0.05% Tween 20 (PBST; Bio-Rad Laboratories Inc.) containing 5% dry skim milk and incubated in PBST with primary antibodies overnight at 4°C and secondary antibodies for 1 hr at RT. Rabbit monoclonal antibodies directed against actin (1:5000; Sigma-Aldrich), and mouse monoclonal antibody directed against HIF-1α (1:1000; BD Biosciences, San Jose, CA, USA). Secondary antibodies were obtained from Invitrogen (Rockville, MD, USA) and Cell Signaling. The blots were scanned the band intensity analysed using ImageJ 1.40 software (National Institutes of Health, Bethesda, MD, USA) [12].

### Reverse transcriptase-polymerase chain reaction (RT-PCR) analysis

Human brain astrocytes were incubated with 1 μM β-lapachone for 4 hrs under co-treatment of 125 μM CoCl_2_ or hypoxia (3% O_2_). RT-PCR was performed and analysed as previously [12]. Total RNA was obtained from cells using the TRI Reagent® (Molecular Research Center, Cincinnati, OH, USA), according to the manufacturer*s instructions. 5 μg of total RNA was converted to first-stranded cDNA using moloney murine leukaemia virus reverse transcriptase and oligo-(dT)^16^ (Invitrogen). Equal amounts of cDNA were subsequently amplified by PCR in a 20-μL reaction volume containing 1× PCR buffer, 300 μM dNTPs, 10 μM specific primer for VEGF (5′-GAGAATTCGGCCTCCGAAACCATGAACTTTCTGT-3′ and 5′-GAGCATGCCCTCCTGCCCGGCTCACCGC-3′), HIF-1α (5′-AGTCGGACAGCCTCAC-3′ and 5′-TGCTGCCTGTATAGGA-3′) and GAPDH (5′-ACCACAGTCCATGCCATCAC-3′ and 5′-TCCACCACCCTGTTGCTGTA-3′) and 1.25 U Taq DNA polymerase (Kapa Biosystems, Woburn, MA, USA). PCR products were separated on 1% agarose gels and visualized using SYBR® Safe DNA gel stain (Invitrogen) under UV transillumination.

### Quantitative real-time PCR (qRT-PCR) analysis

Total RNA was isolated using TRI reagent. Reverse-transcriptase reaction was performed with ImProm II reverse-transcriptase (Promega BioSystems Inc., Seoul, Korea) with oligo-dT priming. qRT-PCR was performed with a TaKaRa Thermal Cycler Dice Real Time System Single TP815 (Takara Bio Inc., Tokyo, Japan) with SYBR Green (Takara Bio Inc.) as fluorescent dyes.

### Qualitative assessment of developmental retinal angiogenesis by immunofluorescence

To evaluate the effect of β-lapachone on physiological angiogenesis of primary vascular plexus in the developmental retina, 1 μM β-lapachone was injected intravitreously C57BL/6J at P4. On P8, when superficial retinal angiogenesis is complete, the eyes were enucleated and fixed in 4% paraformaldehyde for 1 hr. The retinas were dissected and treated with 1% Triton X-100 in PBS (0.1 mM CaCl_2_·H_2_O, 0.1 mM MgCl_2_, 0.1 mM MnCl_2_·4H_2_O, pH 6.8) for 1 hr. Then, the retina was incubated overnight at 4°C with primary antibodies; goat anti-isolectin B4 antibody(1:100; Cell Signaling) and goat anti-type IV collagen polyclonal antibody (1:50; Abcam, Millipore, Bedford, MA, USA). And the retina was incubated with secondary antibody (Alexa Fluoro 488 donkey anti-goat antibody, 1:100; Invitrogen) for 2 hrs. After brief washing with PBS, the retina was flat-mounted and viewed by a fluorescein microscopy (BX50; Olympus) at a magnification of 100×.

### Immunohistochemistry

To evaluate the effect of β-lapachone on physiological retinal angiogenesis in the development, the immunohistochemistry for isolectin B4 and type IV collagen was performed in the retinal cross-section on P8 and P16 after intraocular injection of β-lapachone at P4. The enucleated mouse eyes used for immunohistochemistry were immersion fixed in 4% formalin and subsequently embedded in paraffin. 4-μm-thick serial sections were prepared from paraffin blocks. Sections were deparaffinized and hydrated by sequential immersion in xylene and graded alcohol solutions, and then treated with proteinase K for 5 min. at 37°C and then treated with normal serum obtained from the same species in which the secondary antibody was developed for 10 min. to block non-specific staining. Slides were incubated overnight at 4°C with rabbit polyclonal antibody against anti-goat isolectin B4 (1:50) and anti-goat type IV collagen (1:50). Alexa fluoro 488 donkey anti-goat (1:400) was used as a secondary antibody. The slides were mounted and observed under fluorescence microscope (BX50; Olympus).

### Statistical analysis

Statistical analyses were performed with SPSS software version 20.0 (SPSS Inc., Chicago, IL, USA). *P* < 0.05 was considered to be statistically significant. Figures are depicted as mean ± SD.

## Results

### Intraocular injection of β-lapachone inhibits retinal neovascularization in OIR

**Fig. 1:**
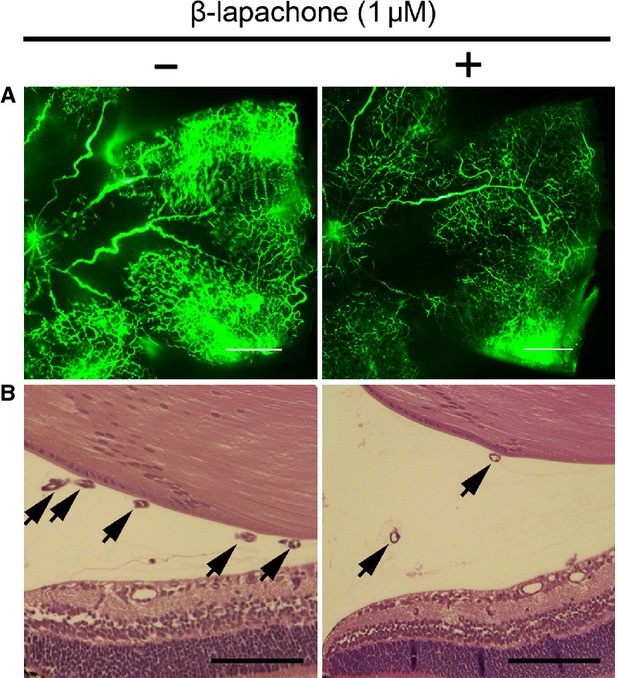
Intraocular injection of β-lapachone inhibits retinal neovascularization in oxygen-induced retinopathy (OIR). (**A**) Retinal vasculatures in control and β-lapachone–treated mice with OIR were evaluated with fluorescein angiography. Whole mount retinal preparation from P17 control and 1 μM β-lapachone intravitreously injected mice of OIR were performed after 1-hr perfusion of fluorescein-conjugated dextran (MW = 500,000). Neovascular tufts of intravitreous neovascularization were observed at the border of vascular and avascular retina; scale bar: 500 μm. (**B**) Vascular lumen was counted for quantitative analysis of retinal neovascularization in OIR. Haematoxylin and eosin–stained cross-sections were prepared from P17 control and 1 μM β-lapachone–treated mice of OIR. Arrows indicate the vascular lumens of new vessels growing into the vitreous. Number of vascular lumens was counted from randomly selected ×40 magnification view; scale bar: 100 μm.

We determined the effect of β-lapachone on retinal neovascularization in OIR mice after intraocular injection of 1 μM β-lapachone at P14. Retinal neovascularization was qualitatively analysed with fluorescein angiography and quantitatively analysed with counting vascular lumen at P17 when retinal neovascularization was peak. Neovascular tufts of intravitreal neovascularization were easily observed at the border of vascular and avascular retina in OIR mice. In contrast, the neovascular tufts in β-lapachone–treated mice were significantly decreased (Fig. 1A). In the vascular lumen analysis, many neovascular lumens were observed in control OIR mice, whereas those were markedly decreased in β-lapachone–treated OIR mice (Fig. 1B). The average number of vascular lumens in β-lapachone–treated OIR mice (10.5 ± 4.5) was significantly decreased than those in control OIR mice (19.0 ± 4.5; *P* < 0.05).

### Intraocular injection of β-lapachone never affects retinal structure nor induces apoptotic cell death in endothelial cells in the retina

**Fig. 2:**
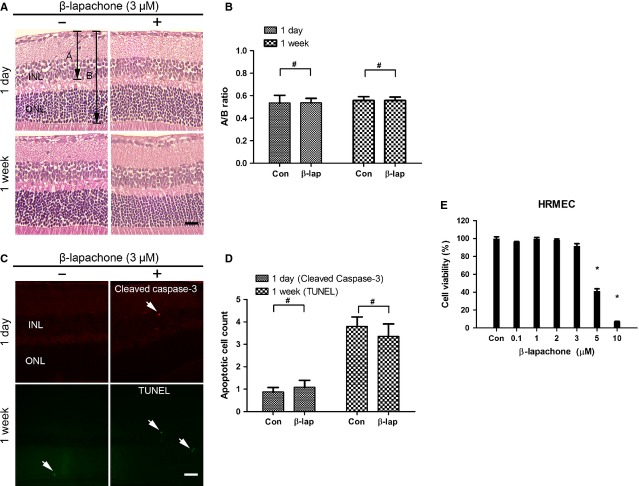
Intraocular injection of β-lapachone never affects retinal structure nor induces apoptotic cell death in endothelial cells in the retina. Retina was evaluated 1 day and 1 week after intravitreous injection of 3 μM β-lapachone respectively. (**A**) Haematoxylin and eosin staining for histological examination was performed. For the evaluation of changes in the retinal layers, the ratio of A (retinal thickness from the internal limiting membrane to the inner nuclear layer) to B (retina thickness from the internal limiting membrane to the outer nuclear layer) was measured at 1 day and 1 week. (**B**) A/B ratio shows no change after β-lapachone both at 1 day and at 1 week. (**C**) Immunofluorescent stains for cleaved caspase-3 (1 day) and TUNEL assay (1 week) were performed to count apoptotic cells at three randomly selected fields (×400) per section. Arrows indicate cleaved caspase-3–positive or TUNEL-positive cells. (**D**) Cleaved caspase-3–positive and TUNEL-positive cells in three fields (×400) were reported as mean ± SEM of at least six eyes at 1 day and 1 week, respectively. (**E**) Effect of β-lapachone on the viability of human retinal microvascular endothelial cells (HRMECs). 1 × 10^4^ HRMECs were seeded into 96-well plates 24 hrs before experiment. Toxicity was assessed by WST-1 proliferation assay 24 hrs after β-lapachone treatment at different concentration (0.1–10 μM) or dimethyl sulfoxide control. The values represent the mean of at least three independent experiments (mean ± SEM). *P < 0.01 (*versus* control), ^#^*P* > 0.05; scale bars: 20 μm. Figures were representative of three independent experiments. INL, inner nuclear layer; ONL, outer nuclear layer.

Retinal toxicity of β-lapachone was evaluated by histological examination with haematoxylin and eosin stain, immunostain with cleaved caspase-3 and TUNEL assay after intraocular injection of 3 μM β-lapachone, which was triple of the therapeutic dose. The retina was normal thickness and all retinal layers were clear without any inflammatory cells in the vitreous, retina, or choroid at 1 and 7 days after injection. The ratios of the retinal thickness from the inner limiting membrane to inner nuclear layer and outer nuclear layer were not significantly changed (Fig. 2A and B). Moreover, there were no increases in cleaved caspase-3–positive cells and TUNEL-positive cells in all retinal layers after intraocular β-lapachone injection at 1 and 7 days respectively (Fig. 2C and D).

Because β-lapachone could have anti-angiogenic activity by inducing apoptotic cell death to endothelial cells in high concentration [17], we performed WST-1 assay with different concentration of β-lapachone (0.1–10 μM) to evaluate the cytotoxicity of β-lapachone on HRMECs. β-lapachone did not affect the cell viability of HRMECs up to 3 μM (Fig. 2E). These data suggested that anti-angiogenic effect of β-lapachone was not by direct cytotoxicity to endothelial cells.

### β-lapachone attenuates VEGF transcription *via* HIF-1α degradation under hypoxic condition in astrocyte

**Fig. 3:**
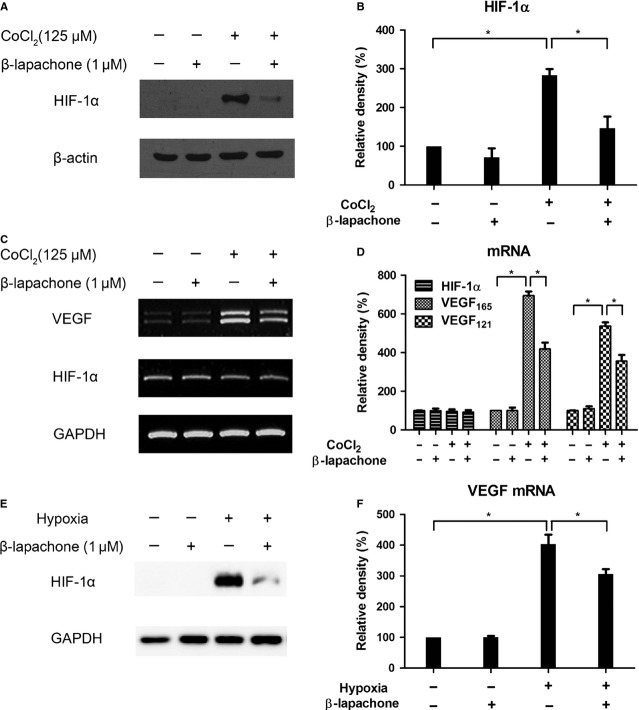
β-lapachone attenuates VEGF transcription *via* HIF-1α degradation under hypoxic condition in astrocyte. Human brain astrocytes were incubated under 125 μM CoCl_2_ treatment (**A**–**D**) or 3% O_2_ hypoxia (**E** and **F**) for 4 hrs with/without co-treatment of 1 μM β-lapachone. (**A**) HIF-1α expression was analysed by Western blot. β-actin served as an internal control. (**B**) Quantitative analysis of HIF-1α was performed by measuring the density relative to the control. (**C**) HIF-1α and VEGF mRNA were analysed by RT-PCR. GAPDH was used as an internal control. (**D**) Semi-quantitative analysis for mRNA was performed by measuring the density relative to the control. (**E**) HIF-1α expression was analysed by Western blot. GAPDH served as an internal control. (**F**) VEGF mRNA was analysed by real-time RT-PCR. GAPDH was used as an internal control. Quantitative analysis for mRNA was performed by measuring the density relative to the control. Each value represents the mean (±SEM) of three independent experiments (*P < 0.05).

Because astrocyte is one of the major sources of VEGF expression in the retina under hypoxia [3,18], we investigated the role of β-lapachone to HIF-1α and VEGF in astrocyte under hypoxic condition. Human brain astrocytes were incubated with CoCl_2_, a hypoxia-mimetic agent, or hypoxia (3% O_2_) for 4 hrs and co-treatment of 1 μM β-lapachone. Like hypoxia, CoCl_2_ can block the degradation and induce the accumulation of HIF-1α protein, a key regulator in the cellular response to hypoxia conditions, and, in turn, up-regulate VEGF transcription. 1 μM β-lapachone reduced CoCl_2_-induced HIF-1α (2.83-fold, *P* = 0.003 to 1.46-fold, *P* = 0.033, Fig. 3A and B). However, β-lapachone did not alter HIF-1α in mRNA level (*P* = 0.701). HIF-1α degradation by β-lapachone led to the decrease in VEGF transcription (VEGF 165: 6.9-fold, VEGF 121: 6.1-fold, *P* < 0.001) to 4.2- and 3.9-fold compared with control respectively (*P* < 0.001; Fig. 3C and D). In addition, 1 μM β-lapachone effectively reduced hypoxia-induced HIF-1α and, in turn, significantly decreased VEGF mRNA from 4.0- to 3.0-fold (*P* < 0.05; Fig. 3E and F).

To determine the effect of β-lapachone on HIF-1a protein synthesis, we examined the accumulation of HIF-1α with the use of proteasome inhibitor MG-132 (10 μM) to prevent HIF-1α degradation in normoxia. HIF-1a rapidly accumulated over a period of 4 hrs in the presence of MG-132 under normoxia. However, accumulation of HIF-1α protein was not impaired in the presence of β-lapachone (Figure S1A). Because MG132 inhibits HIF-1α degradation, the observation that β-lapachone did not decreased HIF-1α accumulation with MG-132 strongly suggests that it promotes HIF-1α degradation rather than interfering its synthesis.

To determine the effect of β-lapachone on the stability of HIF-1 α, we first induced HIF-1α accumulation by exposing the cells to CoCl_2_ to mimic hypoxia condition for 4 hrs together with β-lapachone and/or MG132. In the presence of β-lapachone, HIF-1α levels declined as expected. However, with MG132, β-lapachone did not reduce HIF-1α level (Figure S1B), suggesting that the inhibitory activity of β-lapachone on HIF-1α is mediated through promoting HIF-1α degradation. These data suggested that β-lapachone reduced VEGF transcription *via* HIF-1α protein degradation under hypoxic condition.

### β-lapachone never mitigates physiological retinal angiogenesis in the retinal development

**Fig. 4:**
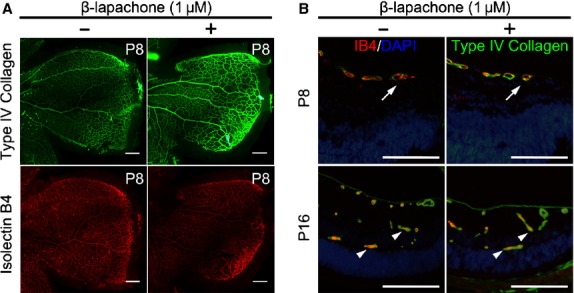
β-lapachone never mitigates physiological retinal angiogenesis in the retinal development. After intraocular injection of 1 μM β-lapachone or 0.1% dimethyl sulfoxide in 1 μl PBS on P4, the effect of β-lapachone on physiological retinal angiogenesis in the developmental retina was evaluated at P8 and P16. (**A**) For primary vascular plexus, whole mount retinal preparation were stained with type IV collagen (green) and isolectin B4 (red); scale bars: 200 μm. (**B**) Retinal cross-sections at P8 and P16 were also evaluated. Retinal vessels were immunostained with type IV collagen (green) and isolectin B4 (red), and nucleus were counterstained with DAPI (blue). Physiological superficial vascular plexus was not regressed by β-lapachone in the peripheral retina at P8. Physiological retinal angiogenesis to intermediate and deep vascular plexus was not affected by β-lapachone at P16. Arrows indicate superficial vessels reaching to the peripheral retina. Arrowheads indicate deep and intermediated plexus; scale bars: 200 μm.

To evaluate the effect of β-lapachone on physiological angiogenesis in the developmental retinal vasculatures, we evaluated the developmental mouse retinal vasculatures after β-lapachone injection on P4. Whole mount retinal preparation from P8 control (0.1% DMSO in PBS) and 1 μM β-lapachone–injected mice were performed. The superficial retinal angiogenesis was not affected by β-lapachone and reached to the periphery of the retina on both control and β-lapachone–injected mice (Fig. 4A). It was also confirmed at the cross-section of the retina on P8 immunostained with isolectin B4 and type IV collagen for retinal vessel (Fig. 4B). Physiological superficial vascular plexus was not regressed by β-lapachone treatment. On P16, when development of intermediate and deep vascular plexuses was complete, physiological retinal angiogenesis to intermediate and deep vascular plexus wase also not affected by β-lapachone treatment (Fig. 4B). Taken together, β-lapachone did not mitigate physiological retinal angiogenesis and induce neurotoxicity in developmental retina.

## Discussion

In this study, we found that β-lapachone inhibited retinal neovascularization in ischaemic retina of OIR. We postulated that the anti-angiogenic effect of β-lapachone came from the reduction in VEGF, which may occur through the reduction in HIF-1α, a transcriptional activator of VEGF in ischaemic retina [19]. Although β-lapachone effectively reduced retinal neovascularization in OIR, the injection of β-lapachone did not affect physiological retinal vascular development in the developing retina and did not induce inflammation or neurotoxicity in the retina. These data suggest that β-lapachone could reduce HIF-1α in hypoxic condition; thus, HIF-1α could be a safe approach to therapy in ROP. The same approach may also be applicable to other ocular neovascularization caused by hypoxia.

VEGF is a major pathogenic factor and therapeutic target in the ocular neovascular diseases. As intravitreal injection of anti-VEGF antibody (bevacizumab) was found to be effective [20], the effectiveness of anti-VEGF antibody (ranibizumab) was determined by two pivotal trials: MARINA [21] and ANCHOR [22]. Recently, the comparison of Age-Related Macular Degeneration Treatment Trials (CATT) revealed equivalent effects on visual acuity after 1 year of monthly injection of anti-VEGF antibodies in neovascular AMD [23]. The anti-VEGF antibodies became the current standard of treatment in neovascular AMD. In addition, intravitreal bevacizumab as an anti-VEGF monotherapy showed a significant benefit for zone I ROP as compared with conventional laser therapy [5]. Although conventional laser therapy led to permanent destruction of the peripheral retina, development of the peripheral retinal vessels continued after treatment with intravitreal bevacizumab.

Despite of the effectiveness of anti-VEGF antibody in ocular neovascular diseases, there were growing bodies of concerns regarding chronic VEGF neutralizing in that VEGF is a survival and neuroprotective factor in retinal neurons [24,25]. Although systemic VEGF blockade did not alter retinal vasculature, it led to increased retinal cell death. Endogenous VEGF has an important role in the maintenance and function of adult retinal neuronal cells [25]. Progressive retinal pigment epithelium (RPE)/photoreceptor atrophy in patients on anti-VEGF therapies was also reported [26]. With regard to the safety concern, direct targeting of VEGF by anti-angiogenic therapy was sufficient to trigger the pathogenesis of thrombotic microangiopathy [27]. In ROP, it is not surprising that peripheral vascular development was disturbed and still remained incomplete after treatment with intravitreal bevacizumab in ROP, because VEGF is not only the pathological angiogenic factor but also important neurotropic and physiological angiogenic factor in prematurity.

Hypoxia inducible factor-1α is one of the major hypoxic response elements in ocular neovascularization. In contrast to the HIF-1α KO mice with laser-induced choroidal neovascularization (CNV), the HIF-1α KO mice with OIR showed no significant difference from the wild-type mice in retinal levels of HIF-1α and VEGF as well as in the number of preretinal neovascular cells [28]. RPE-derived HIF-1α plays a key role in CNV, but not in ischaemia-induced retinal NV. However, this did not suggest that HIF-1α is not important in the pathogenesis of OIR, rather this implied that astrocyte or muller cell–derived HIF-1α is more important than RPE-derived HIF-1α in ischaemia-induced retinal NV. In addition, the elimination of HIF-1α rescued pathological angiogenesis in the laser-induced CNV model without vision loss or endothelial damage in mice, which were observed in VEGF deletion [6]. Normal angiogenic gene expression in HIF mutants suggested that HIFs become activated in pathological states to promote VEGF-mediated neovascularization, but are not required to maintain physiological vasculature [6].

In this regards, HIF-1α, in astrocyte during ROP, is a reasonable target to block in that astrocyte hypoxic response is essential for pathological angiogenesis, not physiological angiogenesis in retina [3]. HIF-1α protein was temporally well correlated with the up-regulated expression of downstream mediators VEGF in OIR [29]. Thus, HIF-1α may be a more desirable target in ocular disease, blocking pathological angiogenesis without damaging healthy vessels, perhaps by modest regulation of endogenous VEGF levels to maintain the function of survival factor. Maintenance VEGF levels at sufficient level for physiological angiogenesis is independent of HIF by several pathways, such as NF-κB/JunB and PGC-1α (peroxisome-proliferator-activated receptor-gamma coactivator-1alpha)/ERRα (estrogen-related receptor-alpha) [30,31]. Moreover, insulin-like growth factor (IGF)-1 partly regulates retinal angiogenesis through the control of p42/44MAPK-induced VEGF activation [32]. Also, IGF-2 contributes to retinal vascularization in ocular development [33]. At least, retinal vessel growth in normal development requires both IGF-1 and VEGF.

Previously, we demonstrated that down-regulation of HIF-1α could inhibit retinal neovascularization in OIR [9]. In addition, Yoshida *et al*. demonstrated that OIR mice with i.p. injections of digoxin showed decrease HIF expression in the eye and resulted in a decrease in retinal NV [34]. In this study, we demonstrated that β-lapachone regulates HIF-1α to inhibit retinal neovascularization without affecting physiological angiogenesis. Although the exact mechanism underlying the β-lapachone–induced degradation of HIF-1α was not elucidated in this study, it is of note that β-lapachone can activate sirtuins, a group of protein deacetylases, by increasing intracellular NAD^+^/NADH ratio [35]. Hypoxia inhibits prolyl hydroxylation, resulting in stabilization of HIF-1α, and through increased ROS, which oxidize PHD-bound iron [36,37]. *In vitro* exposure to CoCl_2_ under normoxic condition produces a hypoxia-mimetic effect with up-regulation of HIF-1α and target gene expression including erythropoietin and VEGF [38,39]. Binding of pVHL, an E3 ubiquitin ligase required for proteasome-dependent HIF-1α degradation, to HIF-1α is dependent on hydroxylation of Pro402 and Pro564 in the ODD domain of HIF-1α through an enzymatic process that requires O_2_ as well as iron and is inhibited by Cobalt Chloride [40–42]. Recently, a group of our collaborators found that sirtuins evoke deacetylation of HIF-1α on specific lysine residues, which in turn enhance prolyl hydroxylation and subsequent ubiquitination of HIF-1α (Gi Ryang Kweon, personal communications). Thus, it is likely that β-lapachone induces proteasome-dependent degradation of HIF-1α through activation of sirtuins.

In conclusion, β-lapachone reduced retinal neovascularization in OIR without toxicity *via* HIF-1 α degradation and subsequent attenuation of VEGF expression. Importantly, β-lapachone–targeting HIF-1α could not mitigate physiological retinal angiogenesis in the retinal development. Taken together, our results suggest that β-lapachone–targeting HIF-1α have a therapeutic potential as an anti-angiogenic drug for variable ischaemia-induced vaso-proliferative retinopathies, especially for ROP.

## Supporting information

**Figure S1** β-lapachone promotes HIF-1α degradation rather than interfering its synthesis. (A) To determine the effect of β-lapachone (1 μM) on HIF-1a protein synthesis, the accumulation of HIF-1α was examined with the use of proteasome inhibitor MG-132 (10 μM) to prevent HIF-1α degradation in normoxia. (B) To determine the effect of β-lapachone on the stability of HIF-1 α, HIF-1α accumulation was examined by exposing the cells to CoCl_2_ (125 μM) to mimic hypoxia condition for 4 hrs together with β-lapachone and/or MG132.
