# Supplementary figures and images for: Beta-lapachone inhibits pathological retinal neovascularization in oxygen-induced retinopathy via regulation of HIF-1α
Source: J Cell Mol Med. 2014 Feb 18;18(5):875–84. doi: 10.1111/jcmm.12235 (PMC4119393; doi:10.1111/jcmm.12235)

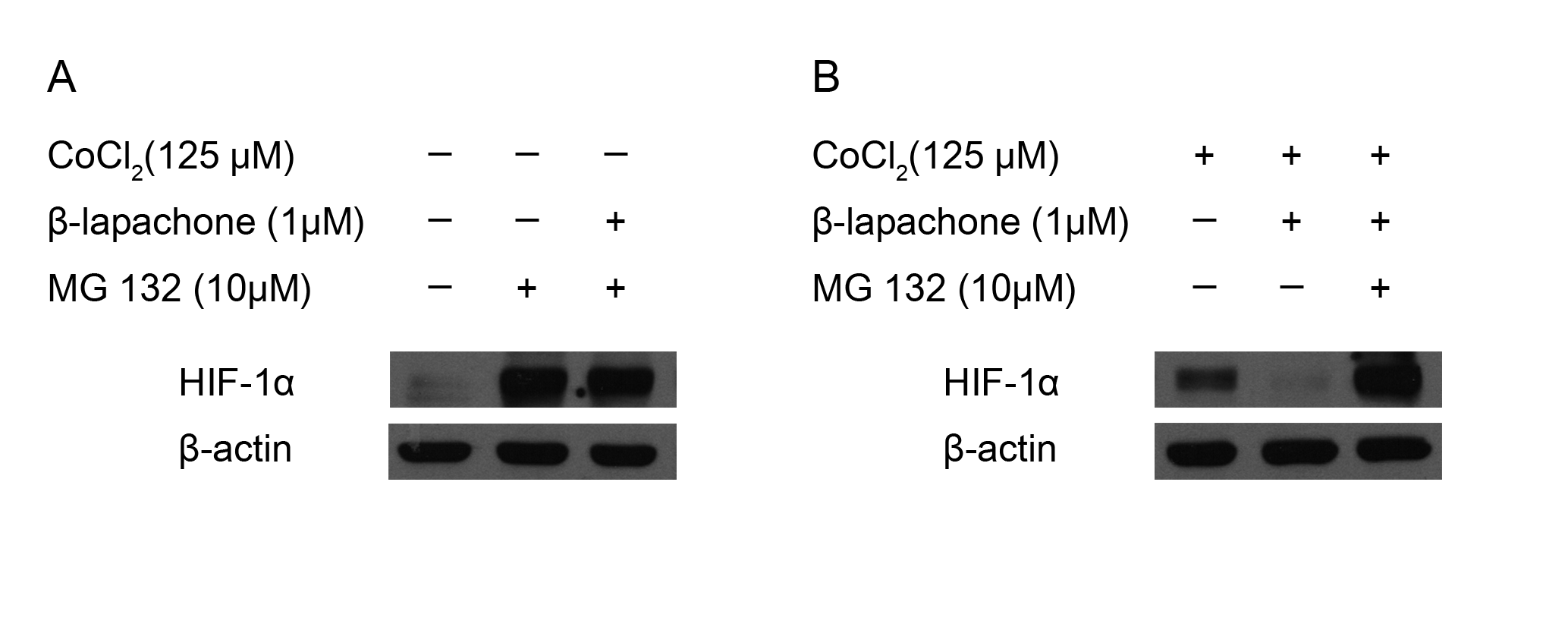

Supplement: Supplementary file 1 — Figure S1 β-lapachone promotes HIF-1α degradation rather than interfering its synthesis. (A) To determine the effect of β-lapachone (1 μM) on HIF-1a protein synthesis, the accumulation of HIF-1α was examined with the use of proteasome inhibitor MG-132 (10 μM) to prevent HIF-1α degradation in normoxia. (B) To determine the effect of β-lapachone on the stability of HIF-1 α, HIF-1α accumulation was examined by exposing the cells to CoCl2 (125 μM) to mimic hypoxia condition for 4 hrs together with β-lapachone and/or MG132. [file jcmm0018-0875-SD1.tif]
